# Clinical Trial Design in Ulcerative Colitis: Interpreting Evolving Endpoints Based on Post Hoc Analyses of the Vedolizumab Phase 3 Trials GEMINI 1 and VISIBLE 1

**DOI:** 10.1093/crocol/otad076

**Published:** 2023-12-18

**Authors:** William J Sandborn, Bruce E Sands, Sharif Uddin, Rana M Qasim Khan, Richa Sagar Mukherjee

**Affiliations:** Division of Gastroenterology, University of California San Diego, La Jolla, CA, USA; Dr. Henry D. Janowitz Division of Gastroenterology, Icahn School of Medicine at Mount Sinai, New York, NY, USA; Takeda Pharmaceuticals U.S.A., Inc., Lexington, MA, USA; Takeda Pharmaceuticals U.S.A., Inc., Lexington, MA, USA; Takeda Pharmaceuticals U.S.A., Inc., Lexington, MA, USA

**Keywords:** ulcerative colitis, clinical trial, Mayo score, regulatory guidance, vedolizumab

## Abstract

**Background:**

The 12-point total Mayo score including a Physician’s Global Assessment (PGA) of disease activity has been used to assess outcomes in clinical trials for ulcerative colitis (UC). In 2016, the US Food and Drug Administration (FDA) issued guidance advising the removal of the PGA in future trials. We examined how endpoints in UC trials have evolved and conducted a post hoc analysis of the GEMINI 1 and VISIBLE 1 trials to understand how the use of a 9-point modified Mayo score, excluding PGA, compares with the total Mayo score.

**Methods:**

Endpoint definitions of clinical remission in phase 3 trials were extracted from published literature and ClinicalTrials.gov. The difference (%Δ) between the proportions of patients in GEMINI 1 and VISIBLE 1 achieving clinical remission with vedolizumab versus placebo at week 52 was measured according to 4 endpoint definitions.

**Results:**

Trials completed up to the end of 2019 used the total Mayo score to assess clinical remission. Most trials that were completed or estimated to be completed by June 2020 or later used modified Mayo scores. Post hoc analysis revealed decreasing endpoint stringency was associated with increasing %Δ values. The modified Mayo score definition most like the definition recommended by the FDA produced %Δ values like those reported using the total Mayo score in GEMINI 1 and VISIBLE 1.

**Conclusions:**

Endpoint definitions for UC clinical trials have evolved following FDA guidance. The efficacy of vedolizumab, measured using modified Mayo scoring, was comparable to values reported using the total Mayo score.

## Graphical Abstract

**Graphical Abstract:**
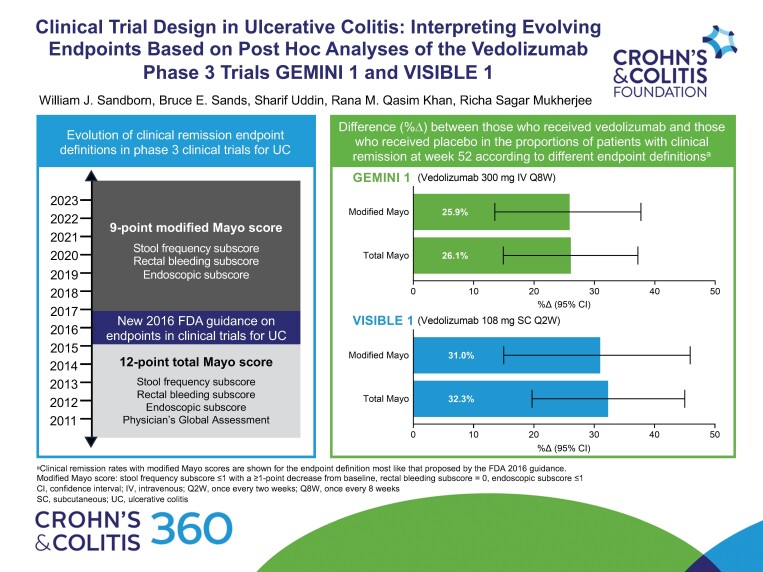

## Introduction

Ulcerative colitis (UC) is a chronic, relapsing–remitting disease characterized by inflammation of the mucosal layer of the colon and rectum that causes damage to the bowel wall. Symptoms include bloody diarrhea, bowel urgency, and abdominal pain.^1^ The introduction of advanced therapies including biologics (eg, anti-tumor necrosis factor α treatments and α4β7 integrin antagonists) and other small molecules (eg, Janus kinase [JAK] inhibitors and sphingosine-1-phophate [S1P] modulators) has enabled dramatic improvements in outcomes for patients with UC. However, some patients experience a primary nonresponse or secondary loss of response to existing advanced therapies, so there remains an unmet need for new improved treatments.^1^ A key to success in trials of new treatments for UC is the ability to accurately measure changes in disease activity. The Mayo score (also known as total Mayo score or complete Mayo score) is a 4-component composite instrument that was developed to measure UC disease activity in clinical trials.^2,3^ It comprises 2 patient-reported outcomes (stool frequency subscore [SFS] and rectal bleeding subscore [RBS]), an endoscopic assessment of the mucosa (endoscopic subscore [ES]), and a Physician’s Global Assessment (PGA) of disease activity, each of which is scored from 0 to 3 to give an overall score ranging from 0 to 12 (higher scores indicate more severe disease).^2^ Since its introduction in 1987, the Mayo score has been used to define clinical remission endpoints in trials of new treatments for patients with moderate-to-severe UC.^4–6^ However, in 2016, the US Food and Drug Administration (FDA) issued guidance recommending changes to the Mayo score for future clinical trials. First, because the presence of friability (even if considered mild by the reader) is not consistent with clinical remission, the FDA recommended that the ES should be modified so that a score of 1 does not include friability. Second, because the factors that the PGA aims to measure are not clearly distinguishable from those measured by the patient-reported SFS and RBS and the objective ES, the FDA recommended its removal.^7^ Therefore, the FDA currently recommends that in developing drugs to treat patients with UC, researchers use a primary endpoint of clinical remission based on a 3-component modified Mayo score that includes the SFS (score ≤1), RBS (score = 0), and ES (score ≤1, modified so that a score of 1 does not include friability) but not the PGA.^7,8^ Note that this modified Mayo score differs from the partial Mayo score, which includes the SFS, RBS, and PGA but does not include the objective ES.^3^

Although there is some evidence that the 3-component modified Mayo score has a strong correlation with the 4-component total Mayo score,^3^ it remains unclear how evolving endpoint definitions affect the measurement of investigational drug efficacy. The phase 3 GEMINI 1 and VISIBLE 1 trials of vedolizumab in UC both began before the new FDA guidance, which makes it challenging to interpret their findings alongside recent trials that may adopt modified Mayo scoring. We have examined how clinical remission endpoint definitions in phase 3 clinical trials in UC have evolved in response to FDA guidance, and conducted post hoc analyses of the GEMINI 1 and VISIBLE 1 trials to evaluate how newer modified Mayo score-based definitions may influence the measurement of efficacy compared with the total Mayo score.

## Methods

### Endpoint Definition Search

To identify relevant clinical trials, ClinicalTrials.gov was searched using the term “ulcerative colitis” and results were filtered to include only active (recruiting and not recruiting) or completed phase 3 interventional clinical trials that enrolled adult patients. Results of initial searches were subsequently manually screened to identify studies that met the following criteria: was a trial of maintenance therapy for patients with moderate-to-severe UC; included a primary (or high-ranked secondary) endpoint of clinical remission (that included details of the endpoint definition); was a trial of advanced biologic or small-molecule therapy (adalimumab, etrasimod, etrolizumab, filgotinib, golimumab, guselkumab, infliximab, mirikizumab, obefazimod, ontamalimab, ozanimod, risankizumab, tofacitinib, upadacitinib, ustekinumab, vedolizumab) versus placebo; was a study based in the United States or a global study with sites in the United States. If required, further details on endpoint definitions were also extracted from primary published data associated with the identified trials (obtained via searching PubMed) and other relevant websites (eg, press releases).

### Post Hoc Analysis

Post hoc analyses were performed using data from the vedolizumab GEMINI 1 (NCT00783718) and VISIBLE 1 (NCT02611830) phase 3 clinical trials.^6,9^ The difference in the proportion of patients who had clinical remission at week 52 between those who received vedolizumab and those who received placebo (treatment difference or %Δ) was measured using 4 endpoint definitions. The chosen endpoint definitions were based on findings from the prior literature search and reflected 3-component modified Mayo score-based endpoints ranging from most (A) to least stringent (D) – A: SFS ≤1 with a ≥1-point decrease from baseline, RBS = 0, ES = 0; B: SFS ≤1, RBS = 0, ES = 0; C: SFS ≤1 with a ≥1-point decrease from baseline, RBS = 0, ES ≤1; D: SFS ≤1, RBS = 0, ES ≤1. The treatment differences with definitions A to D were compared with the definitions used in the original clinical trials (E: total Mayo score at week 52 ≤2 with no individual subscore >1).

## Results

### Evolving Endpoints

The initial search of ClinicalTrials.gov identified 145 phase 3 clinical trials in patients with UC. Following manual screening, 19 studies met the eligibility criteria. Except for the True North study, which began in June 2015, all identified trials that began up to, and including, December 2015 used definitions of clinical remission based on the 4-component total Mayo score that included the PGA (Table 1). All trials that began after the publication of updated FDA guidance in August 2016 used a 3-component modified Mayo score-based definition of clinical remission (which excluded the PGA). No trial completed before March 2020 used the modified Mayo score (Table 1). Most trials that adopted a modified Mayo score-based definition used SFS ≤1, RBS = 0, and ES ≤1. Some differences were observed among definitions relating to how friability was assessed as part of the ES (Table 1).

**Table 1.** Endpoint definitions of clinical remission during the maintenance treatment component of phase 3 UC trials.

Advanced therapy name	Mechanism of action	Trial name (ClinicalTrials.gov number)	Trial start date	Trial completion date	Endpoint definition (clinical remission, maintenance phase)
Obefazimod	miR-124 regulator	ABTECT Maintenance (NCT05535946)	Jan 2023	Jun 2025^a^	Modified Mayo score at week 44.^b^ SFS ≤1, RBS = 0, and ES ≤1 (friability is scored as 2)
Guselkumab	IL-23 antagonist	QUASAR (NCT04033445)	Sep 2019	Oct 2027^a^	Modified Mayo score at week 44.^b^ SFS ≤1 (with no increase from baseline), RBS = 0, and ES ≤1 (excluding friability)
Etrasimod	S1P receptor modulator	ELEVATE UC 52 (NCT03945188)	Jun 2019	Feb 2022	Modified Mayo score at week 52. SFS = 0 (or = 1 with a ≥1-point decrease from baseline), RBS = 0, and ES ≤1 (excluding friability)
Mirikizumab	IL-23 antagonist	LUCENT 2 (NCT03524092)	Oct 2018	Apr 2026^a^	Modified Mayo score at week 40.^c^ SFS ≤1 (with ≥1-point decrease from baseline), RBS = 0, and ES ≤1 (excluding friability)
Risankizumab	IL-23 antagonist	M16-066 (NCT03398135)	Aug 2018	May 2024^a^	Modified Mayo score at week 52. SFS ≤1 (with no increase from baseline), RBS = 0, and ES ≤1 (excluding friability)
Ontamalimab	anti-MAdCAM-1	FIGARO UC 303 (NCT03290781)	Apr 2018	Jul 2021	Modified Mayo score at week 52. SFS ≤1 (with a ≥1-point decrease from baseline), RBS = 0, and ES ≤1 (modified, excluding friability)
Filgotinib	JAK inhibitor	SELECTION (NCT02914522)	Nov 2016	Mar 2020	Modified Mayo score at week 58. SFS ≤1 (with a ≥1-point decrease from baseline), RBS = 0, and ES ≤1
Upadacitinib	JAK inhibitor	U-ACHIEVE (NCT02819635)	Sep 2016	Dec 2021	Modified Mayo score at week 52. SFS ≤1 and not greater than baseline, RBS = 0, and ES ≤1 (includes friability^d^)
Vedolizumab	α4β7 integrin antagonist	VISIBLE 1 (NCT02611830)	Dec 2015	Aug 2018	Total Mayo score at week 52 ≤2 and no individual subscore >1
Ustekinumab	IL-12/IL-23 antagonist	UNIFI (NCT02407236)	Jul 2015	Nov 2021	Total Mayo score at week 44^b^ ≤2 and no individual subscore >1
Vedolizumab	α4β7 integrin antagonist	VARSITY (NCT02497469)	Jun 2015	Jan 2019	Total Mayo score at week 52 ≤2 and no individual subscore >1
Ozanimod	S1P receptor modulator	True North (NCT02435992)	Jun 2015	Jun 2020	Modified Mayo score at week 52. SFS ≤1 (with a ≥1-point decrease from baseline), RBS = 0, and ES ≤1
Etrolizumab	α4β7 integrin antagonist	LAUREL (NCT02165215)	Aug 2014	Apr 2020	Total Mayo score at week 62 ≤2, RBS = 0, and no individual subscore >1
Etrolizumab	α4β7 integrin antagonist	HICKORY (NCT02100696)	May 2014	Apr 2020	Total Mayo score at week 66 ≤2, RBS = 0, and no individual subscore >1
Tofacitinib	JAK inhibitor	OCTAVE Sustain (NCT01458574)	July 2012	May 2016	Total Mayo score at week 52^e^ ≤2, RBS = 0, and no individual subscore >1
Vedolizumab	α4β7 integrin antagonist	GEMINI 1 (NCT00783718)	Jan 2009	Mar 2012	Total Mayo score at week 52 ≤2 and no individual subscore >1
Golimumab	anti-TNFα	PURSUIT-M (NCT00488631)	Sep 2007	Feb 2015	Total Mayo score at weeks 30 and 54 ≤2 and no individual subscore >1
Adalimumab	anti-TNFα	ULTRA 2 (NCT00408629)	Nov 2006	Mar 2010	Total Mayo score at week 52 ≤2 and no individual subscore >1
Infliximab	anti-TNFα	ACT 1 (NCT00036439)	Feb 2002	Jan 2007	Total Mayo score at week 54 ≤2 and no individual subscore >1

Modified Mayo score excludes PGA. Total Mayo score includes PGA.

Abbreviations: ES, endoscopic subscore; IL, interleukin; JAK, Janus kinase; MAdCAM, mucosal addressin cell adhesion molecule; miR, micro-RNA; PGA, Physician’s Global Assessment; RBS, rectal bleeding subscore; S1P, sphingosine-1-phosphate; SFS, stool frequency subscore; TNF, tumor necrosis factor; UC, ulcerative colitis.

^a^Estimated completion date according to ClinicalTrials.gov (accessed May 16, 2023).

^b^Eligible patients will already have completed 8 weeks of induction therapy, so week 44 is equivalent to week 52 in trials that combine the induction and maintenance parts.

^c^Eligible patients will already have completed 12 weeks of induction therapy, so week 40 is equivalent to week 52 in trials that combine the induction and maintenance parts.

^d^Evidence of friability during endoscopy in participants with otherwise mild endoscopic activity conferred an endoscopic subscore of 2.

^e^Eligible patients will already have completed 8 weeks of induction therapy, so week 52 is equivalent to week 60 in trials that combine the induction and maintenance parts.

### Post Hoc Vedolizumab Efficacy Assessment

**Figure 1.**
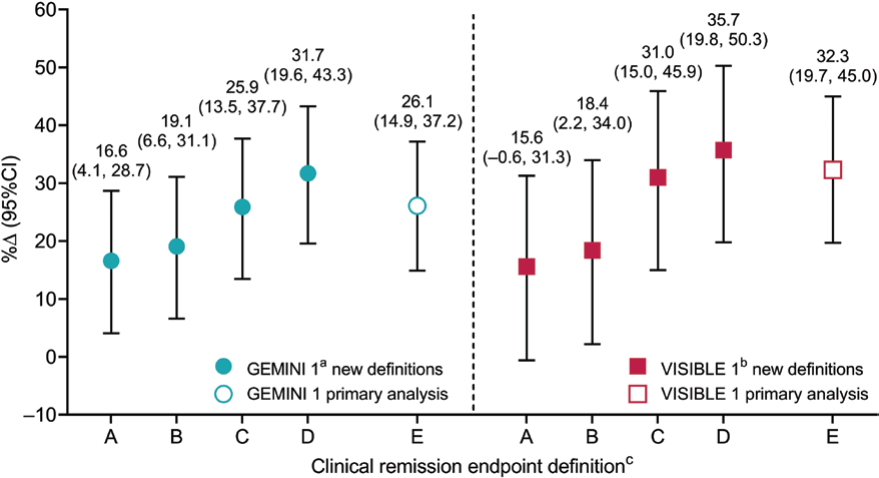
Difference in the proportion of patients with clinical remission at week 52 (%Δ) between those who received vedolizumab and those who received placebo in the GEMINI 1 and VISIBLE 1 trials according to 4 endpoint definitions of differing stringency and the endpoint definitions used in the primary analyses. 95% CIs were calculated based on the exact method. ^a^Patients received intravenous vedolizumab once every 8 weeks. ^b^Patients received subcutaneous vedolizumab once every 2 weeks. ^c^New definitions are based on most (A) to least (D) stringent criteria. A, SFS ≤1 with a ≥1-point decrease from baseline, RBS = 0, ES = 0. B, SFS ≤1, RBS = 0, ES = 0. C, SFS ≤1 with a ≥1-point decrease from baseline, RBS = 0, ES ≤1. D, SFS ≤1, RBS = 0, ES ≤1. E, Total Mayo score at week 52 ≤2 with no individual subscore >1. CI, confidence interval; ES, endoscopic subscore; RBS, rectal bleeding subscore; SFS, stool frequency subscore.

In total, 410 patients with UC from the GEMINI 1 (*N* = 248) and VISIBLE 1 (*N* = 162) trials were included. As previously reported, baseline patient characteristics and demographics were similar for patients who received vedolizumab and those who received a placebo in both trials.^6,9^ The treatment differences measured using endpoint definitions of differing stringency are shown for the GEMINI 1 trial (placebo, *n* = 126; vedolizumab 300 mg intravenously once every 8 weeks, *n* = 122) and the VISIBLE 1 trial (placebo, *n* = 56; vedolizumab 108 mg subcutaneously once every 2 weeks, *n* = 106) in Figure 1. For both GEMINI 1 and VISIBLE 1, there was a trend of increasing treatment difference values as the stringency of the endpoint definition decreased. Endpoint definition C (SFS ≤1 with a ≥1-point decrease from baseline, RBS = 0, ES ≤1) produced treatment differences most like those found using the total Mayo score-based definition (Figure 1, definition E).

## Discussion

In 2016, the US FDA issued new guidance on definitions of clinical remission for use as an endpoint in clinical trials in UC. Because of this guidance, endpoint definitions have evolved. In our review of endpoint definitions, we found that all phase 3 trials of investigational drugs aiming for approval in the United States that began in 2016 or later adopted a 3-component modified Mayo score-based definition for their clinical remission endpoints. In our post hoc analyses, the endpoint definitions that were like those most recently recommended by the FDA^8^ and those used in more recent clinical trials (Table 1) were the 2 least stringent that were tested (C and D). Both of these definitions produced treatment differences that were similar to those previously reported using the total Mayo score in the GEMINI 1 and VISIBLE 1 trials. Thus, our findings show that the efficacy of both intravenous and subcutaneous vedolizumab is similar when measured using the newer modified Mayo score-based definitions and the total Mayo score. The similarity among the outcomes measured with modified Mayo scores and total Mayo scores in patients with UC in our analysis also supports previous findings that suggested a strong correlation between these 2 instruments.^3^ Similar studies with other biologics in patients with UC are also supported by our findings. In the UNIFI study of ustekinumab in patients with moderate-to-severe UC, the primary endpoint during the maintenance phase of the trial was clinical remission based on the total Mayo score. However, the study also contained an alternative primary endpoint that used a modified Mayo score, excluding the PGA. The proportion of patients who had clinical remission at week 52 was 43.8% (ustekinumab 90 mg once every 8 weeks) when the total Mayo score was used and 42.6% when the modified Mayo score was used.^10^ In a post hoc analysis of data from the OCTAVE program, clinical remission at week 52, defined by total Mayo score, was observed in 40.6% of patients who received tofacitinib (10 mg twice daily) compared with 42.1% when a modified Mayo score was used.^11^

Furthermore, the lack of notable discrepancies between treatment differences measured using an instrument including the PGA and one excluding the PGA suggests that the PGA, when performed by an experienced gastroenterologist, can provide a reliable assessment of disease activity. The PGA may, therefore, continue to have use in clinical practice or as a component of the partial Mayo score, in which it can be used as an interim assessment of disease activity in clinical trials when endoscopic data are not available. Overall, our findings suggest that comparisons of clinical remission endpoints can be made between older trials that used the total Mayo score, including the PGA, and newer trials using modified Mayo score-based endpoint definitions.

## Data Availability

The data sets, including the redacted study protocol, redacted statistical analysis plan, and individual participants’ data supporting the results reported in this article, will be made available within 3 months from initial request to researchers who provide a methodologically sound proposal. The data will be provided after their de-identification, in compliance with applicable privacy laws, data protection, and requirements for consent and anonymization.

## Conference Presentation

These data were previously presented at the American College of Gastroenterology 2021 Annual Scientific Meeting & Postgraduate Course, October 22–27, 2021, Las Vegas, NV, USA. Poster P1659.
